# Supplementary material for: Modeling glioblastoma heterogeneity as a dynamic network of cell states
Source: Mol Syst Biol. 2021 Sep 16;17(9):e10105. doi: 10.15252/msb.202010105 (PMC8444284; doi:10.15252/msb.202010105)
Supplement: Supplementary file 6 — Source Data for Figure 5 [file MSB-17-e10105-s004.zip › Figure5A_sourcedata/GSEA_3071/hallmarks_stateII.GseaPreranked.1623742536692/HALLMARK_INTERFERON_ALPHA_RESPONSE.html]

Details for gene set HALLMARK\_INTERFERON\_ALPHA\_RESPONSE[GSEA]

|  || Dataset | state23071 |
| Phenotype | NoPhenotypeAvailable |
| Upregulated in class | na\_neg |
| GeneSet | HALLMARK\_INTERFERON\_ALPHA\_RESPONSE |
| Enrichment Score (ES) | -0.23814923 |
| Normalized Enrichment Score (NES) | -0.9396293 |
| Nominal p-value | 0.5359116 |
| FDR q-value | 0.71702355 |
| FWER p-Value | 1.0 |
Table: GSEA Results Summary

  

Fig 1: Enrichment plot: HALLMARK\_INTERFERON\_ALPHA\_RESPONSE      
 Profile of the Running ES Score & Positions of GeneSet Members on the Rank Ordered List

  

| PROBE | GENE SYMBOL | GENE\_TITLE | RANK IN GENE LIST | RANK METRIC SCORE | RUNNING ES | CORE ENRICHMENT || 1 | PNPT1 |  |  | 728 | 0.523 | -0.1173 | No |
| 2 | PLSCR1 |  |  | 992 | 0.446 | -0.1295 | No |
| 3 | IRF1 |  |  | 1173 | 0.403 | -0.1265 | No |
| 4 | MOV10 |  |  | 1629 | 0.319 | -0.2007 | Yes |
| 5 | PSMA3 |  |  | 1692 | 0.312 | -0.1794 | Yes |
| 6 | TRIM14 |  |  | 1744 | 0.305 | -0.1561 | Yes |
| 7 | PROCR |  |  | 1757 | 0.304 | -0.1234 | Yes |
| 8 | SLC25A28 |  |  | 1759 | 0.304 | -0.0881 | Yes |
| 9 | RIPK2 |  |  | 1892 | 0.286 | -0.0869 | Yes |
| 10 | TXNIP |  |  | 1918 | 0.283 | -0.0599 | Yes |
| 11 | CASP1 |  |  | 1994 | 0.273 | -0.0463 | Yes |
| 12 | TRIM26 |  |  | 2005 | 0.272 | -0.0169 | Yes |
| 13 | EIF2AK2 |  |  | 2148 | 0.257 | -0.0215 | Yes |
| 14 | ISG15 |  |  | 2397 | -0.270 | -0.0507 | Yes |
| 15 | PSME1 |  |  | 2413 | -0.272 | -0.0225 | Yes |
| 16 | CNP |  |  | 2457 | -0.278 | -0.0004 | Yes |
| 17 | LPAR6 |  |  | 2622 | -0.304 | -0.0050 | Yes |
| 18 | PARP9 |  |  | 2780 | -0.332 | -0.0046 | Yes |
| 19 | IRF2 |  |  | 2830 | -0.341 | 0.0234 | Yes |
| 20 | IRF9 |  |  | 2901 | -0.356 | 0.0479 | Yes |
| 21 | MVB12A |  |  | 2938 | -0.362 | 0.0815 | Yes |
| 22 | STAT2 |  |  | 3496 | -0.539 | 0.0080 | Yes |
| 23 | IFITM3 |  |  | 3578 | -0.589 | 0.0572 | Yes |
| 24 | GMPR |  |  | 3590 | -0.599 | 0.1247 | Yes |
Table: GSEA details [plain text format]

  

Fig 2: HALLMARK\_INTERFERON\_ALPHA\_RESPONSE: Random ES distribution      
 Gene set null distribution of ES for **HALLMARK\_INTERFERON\_ALPHA\_RESPONSE**
